# Phlebotomy for rapid weaning and extubation in COPD patient with secondary polycythemia and respiratory failure

**DOI:** 10.4103/0970-2113.59264

**Published:** 2010

**Authors:** Swagata Tripathy, Sudhansu S. Panda, Biswajit Rath

**Affiliations:** *Department of Kalinga Institute of Medical Sciences, Bhubaneswar, India*; 1 *Department of Internal Medicine, Kalinga Institute of Medical Sciences, Bhubaneswar, India*; 2 *Department of Cardiology, Kalinga Institute of Medical Sciences, Bhubaneswar, India*

**Keywords:** Hyperviscosity complications, phlebotomy, secondary polycythemia, ventilator dependence, weaning

## Abstract

The increased incidence of ventilator-associated complications in patients with chronic obstructive pulmonary disease (COPD) necessitates rapid weaning and extubation. The presence of secondary polycythemia in this subgroup increases the incidence of stroke and myocardial infarction due to hyperviscosity and tissue hypoxia. We present a 58-year-old male patient of COPD with secondary polycythemia (hematocrit 64%) who had possible hyperviscosity-related complications leading to cardiac arrest after a minor surgical procedure. The patient developed ventilator dependence after recovery. Phlebotomy was done to remove 10% of total blood volume. Symptomatic improvement was dramatic. Improvement in weaning indices like rapid shallow breathing index and PaO_2_/PAO_2_ was observed facilitating rapid weaning and early extubation. Monitored, acute phlebotomy is safe and cost-effective. It decreases blood volume and viscosity, increases cardiac output and improves exercise tolerance in patients.

## INTRODUCTION

Acute episodes of respiratory failure in patients with chronic obstructive pulmonary disease (COPD) caused by infections, heart failure, pulmonary embolism or other causes account for 5-10% of emergency medical admissions to a hospital.[1] Invasive mechanical ventilation often initiated in this scenario has a number of complications,[2] particularly when prolonged. COPD accounts for approximately 25% of weaning failure defined as those still ventilator-dependent three weeks or more after recovery from the condition precipitating ICU admission.[1]

Secondary polycythemia, a complication of chronic hypoxia in COPD, results in tissue hypoxia, metabolic acidosis, increased thrombogenocity and higher incidence and severity of coronary disease and stroke in these patients.[3]

We present a patient with COPD and secondary polycythemia who developed ventilator dependence, post-resuscitation, from complications after a minor surgical procedure. Weaning and extubation were facilitated with phlebotomy removing 10% of his blood volume. To the best of our knowledge this is the only report on the use and benefits of phlebotomy for this indication. We discuss the work done in this area and possible causes of dramatic improvement seen in our patient.

## CASE REPORT

A 58-year-old male presented in the month of May with scrotal swelling and cellulitis. Patient was a chronic smoker (over 200 pack years) diagnosed as COPD with corpulmonale. He had a ruddy complexion, finger tip cyanosis and injected conjunctiva. He complained of easy fatigability, headaches, daytime somnolence and dyspnoea on exertion (elicited retrospectively) pointing to polycythemia related hyperviscosity. Previous pulmonary function test revealed severe restriction and very severe obstruction (FVC, FEV_1_ and PEF of 34, 14 and 17 percent predicted respectively).

Routine investigation revealed hemoglobin of 19% and hematocrit of 64% [Table 1]. Total leucocyte and platelet count, liver and renal function and chest roentgenogram was normal. Electrocardiogram (ECG) showed evidence of p pulmonale. On the day of the procedure, patient's heart rate was 75/min, blood pressure 116/78 mm Hg, respiratory rate of 16/min; chest was clear on auscultation. Patient was only taking his regular dose of deriphyllin. Pre-operative blood gas analysis was not done. A bedside incision and drainage of swelling was done after local infiltration of 10 ml one per cent Lignocaine. Sedation and parenteral opiod analgesia was avoided in view of COPD.

**Table 1:** Hematologic parameters and weaning outcomes

Day	1	2	3	5	10
Hemoglobin gm/dl	19.1	19.0	18.8	16.8	16.0
TRBC 10^6^/μl	6.78	6.75	6.81	6.10	5.84
Hematocrit % wintrobs	65	64	62.4	56.2	55
Phlebotomy on day 3 (48 hrs after ventilation)
Weaning index	Previous	Two hrs after phlebotomy
fR breaths/min	34	26
VT mL	320	340
PaO_2_/PAO_2_	0.35	0.46
Cdyn mL/cmH_2_O	22	24
fR/VT breaths/min/L	106	76

fR: Respiratory frequency; VT: Tidal volume; PaO_2_: Arterial oxygentension; PAO_2_: Alveolar oxygen tension; Cdyn effective compliance of there spiratory system

Towards the end of an otherwise uneventful procedure (15 minutes) patient started complaining of respiratory distress. Chest auscultation revealed distant respiratory sounds and diffuse rhonchi. Room air saturation was now monitored to be 82%. Patient was shifted to the Intensive Care Unit (ICU), 20 minutes later, for emergency management after oxygen at 10 liters/min via a face mask and salbutamol nebulization failed to improve his condition.

In the ICU invasive ventilation was initiated in view of life threatening acidemia (pH 7.001) and hypercarbia (PaCO_2_ 150 mmHg) after a trial of non-invasive ventilation (NIV) for 45 minutes which failed to improve his blood gas picture [Table 2]. Medical management was instituted for hyperkalemia (6.7 m Eq/dl). Serum cardiac enzymes and D-Dimer levels were within normal limits. Ischemic changes in ECG, generalized seizures and cardiac arrest followed in succession. The patient was defibrillated twice and revived.

**Table 2:** Serial blood gases

Hour	0	1	4	6	10	24	60
pH	7.057	7.001	7.063	7.114	7.25	7.40	7.42
PaO_2_ mmHg	166.9	109.5	65.0	72	70	87.0	65
PaCO_2_ mmHg	148	150	112	80	60	47.6	50
HCO_3_ mmHg	34	35	33	31	30	28	29
BE	−3.6	−4.1	−4.3	−4.0	−3	4.4	4.1
K^+^	6.5	-	6.5		6.2	3.4	2.9

Noninvasive ventilation started at 0 hr. IV started by 1 hr. Cardiac arrest at 8 hr. Weaning started by 30 hrs. First phlebotomy at 40 hrs, extubation at 50 hrs

Over the next few hours his condition stabilized. His blood sample tended to rapid clumping pointing towards hyperviscosity-antiplatelets and antithrombotics were started. His general condition improved after hydration, steroids, antibiotics and physiotherapy. Chest was clear on auscultation. Major criteria for weaning being satisfied, weaning efforts were started with an aim at early separation from ventilator. Patient developed tachypnoea, increased accessory muscle activity, diaphoresis, facial signs of distress, tachycardia, arrhythmias and hypotensio. SaO_2_ and/or the pH fell below 88% and 7.3, respectively with weaning attempts (Synchronised Intermittent Mandatory Ventilation with Pressure Support mode on *Macquet servo i* ventilator).

Associating signs of dependence to tissue hypoxia due to hyperviscosity phlebotomy was done removing total 10% blood volume in two sittings, four hours apart, on the third day. After the phlebotomy he claimed to be “feeling my fittest in many years”. Extubation after four hours was successful. There was no requirement for non-invasive ventilation after extubation. There was no significant improvement in pulmonary functions tests however. He was discharged with advice of intermittent oxygen therapy and follow-up after seven days.

## DISCUSSION

We postulate a combination of factors for the sudden decompensation of our patient. The stress of surgery and imperfect pain relief with local anesthesia may have caused tachypnoea. Increased respiratory rate increased the dead space ventilation and as expiratory time shortened, further muscle loading resulted, causing additional dynamic hyperinflation. Increased pulmonary vascular resistance and reduced venous return was exacerbated further by the supine position, impaired right heart function and decreased cardiac output. Inadequate systemic oxygen delivery aggravated by the hyperviscosity then added a metabolic component to the respiratory acidosis. There is a logarithmic rise in blood viscosity beyond a hematocrit of 55%. Sludging of blood in capillaries causes tissue hypoxia, metabolic acidosis, increased thrombogenocity and higher incidence and severity of coronary disease and stroke in these patients[3] as seen in ours. Hypoxaemia and acidosis further impair respiratory muscle function.

A diagnosis of secondary polycythemia was made in our patient based on well-defined criteria of British Committee for Standards in Hematology.[4] Management of secondary polycythemia is traditionally done with long-term oxygen administration. Phlebotomy has a doubtful role (Grade B level III) as the erythrocytosis is considered a response to hypoxia.[4] Dayton and colleagues[5] noted that in patients with severe chronic lung disease and secondary polycythemia, phlebotomy produced subjective benefit in the majority; this improvemt seemed to be more dramatic in those with evidence of CHF and an initial hematocrit reading greater than 60%. This improvement is probably primarily effected by blood viscosity reduction, although blood volume reduction may play a role in those patients showing an immediate response. Today, classical blood letting has been replaced by erythrapheresis. After erythrapheresis appreciable symptomatic improvement and increases in exercise tolerance and mental alertness has been seen.[6]

Among the common pathophysiological factors associated with failure to wean[7] are those related to central drive (sedation, analgesia or anesthesia, coma, raised intra-cranial pressure, hypercapnia), respiratory muscle strength (hypophosphataemia, disuse atrophy, sepsis, polyneuropathy/myopathy), and load applied to the muscles (hyperinflation, left ventricular failure, bronchospasm, lung fibrosis). We suggest a combination of hyperinflation and increased work of breathing due to hyper-viscosity related decreased oxygen supply as a cause of difficult weaning in our patient, which improved with phlebotomy.

We recorded remarkable improvement-decrease in respiratory rate and improvement in PaO_2_/PAO_2_ ratio occurred after phlebotomy-other parameters remaining almost same [Table 1]. After each phlebotomy patient felt better and maintained 100% saturation on room air (previously 90-91%) up to eight hours. Subjective feeling of improvement lasted for a longer duration of time and a previously anxious patient who was resenting any attempts at weaning started to demand extubation within a few hours.

Phlebotomy in patients with secondary polycythemia has been shown to improve cerebral blood flow,[8] specially supra-tentorial[9] and bring an improvement in subjective wellbeing. Decrease in incidence and severity of angina pectoris[10] and improvement in exercise tolerance has been observed after phlebotomy.[11] Data tends to support the hypothesis that the afterload on the left ventricle is reduced resulting in improved myocardial contractility and left ventricular function. Improved peripheral oxygen uptake may also be a factor.[12]

Follow-up pulmonary function tests did not show a significant improvement in keeping with the conclusions of Dayton *et al.* Improvement in oxygenation, seen in our patient, may be attributable to a mild congestion not appreciated clinically or radiographically which improved with volume reduction. The decrease in respiratory rate may be explained by a decreased work of breathing due to increased cardiac output and oxygen delivery explaining the marked subjective improvement the patient claimed.

Our experience would highlight the benefits of bedside phlebotomy, which is readily available, cost- effective and safe as a one-time procedure to facilitate early weaning and extubation in difficult-to-wean COPD patients with secondary polycythemia. This may be tried prior to extubation to avoid need for/improve chances of success of NIV ventilation for weaning. We would also like to stress on the possible benefit of monitored anesthesia care even for minor surgical procedures in these patients.
